# The Evidence-Based Application of Spinal-Hip Biomechanics Research Among Saudi Physiotherapists: A Cross-Sectional Study

**DOI:** 10.7759/cureus.97650

**Published:** 2025-11-24

**Authors:** Raee S Alqhtani

**Affiliations:** 1 Medical Rehabilitation Department-Physiotherapy Program, Applied Medical Science College, Najran University, Najran, SAU

**Keywords:** biomechanics, evidence-based practice, hip, lumbar, physiotherapists

## Abstract

Background and objective

Evidence-based practice (EBP) in Saudi Arabia remains underexplored, particularly in physiotherapy. While recent studies have acknowledged its significance, limited attention has been paid to the integration of contemporary spinal biomechanics research into clinical practice. It also remains unclear whether physiotherapy practitioners in Saudi Arabia are effectively applying emerging insights on spinal-hip biomechanics, despite their potential to improve rehabilitation outcomes. This study, therefore, sought to assess physiotherapists’ perceptions of spinal-hip biomechanics research, evaluate its use in clinical settings, and identify barriers to its adoption in Saudi Arabia.

Methods

A self-administered, cross-sectional, questionnaire-based survey was conducted. The instrument consisted of 23 items organized across seven sections and was distributed to physiotherapy practitioners across 13 regions in Saudi Arabia. A total of 64 physiotherapists participated. Recruitment and survey promotion were facilitated through social media platforms (Facebook, Twitter) and WhatsApp*. *

Results

Male physiotherapists constituted the majority of participants, with the Asir region showing the highest participation rate (17.2%) and four regions reporting the lowest (3%). Female respondents provided higher affirmative responses than males on all items except their ability to read, critique, and summarize research, where their scores were 7% lower. Regarding spinal motion assessment and measurement methods, underestimated responses were common among both genders, except for one item in which 59.5% of male participants responded affirmatively.

Conclusions

Saudi physiotherapists largely support EBP principles, yet fewer than one-third of institutions provide free access to research databases. This institutional shortfall restricts the translation of evidence into practice, impedes the adoption of EBP, and may adversely impact professional development and patient care.

## Introduction

Spinal mobility is fundamental to both occupational tasks and activities of daily living. On average, sagittal plane spinal movements occur approximately 4,400 times within 24 hours, with two-thirds ranging between 5° and 10° [1]. Lumbar dysfunction remains one of the most prevalent musculoskeletal conditions requiring rehabilitation and is a leading cause of functional impairment. Understanding the regional distribution of spinal motion across anatomical planes and the relative contribution of thoracolumbar segments is essential for accurate spinal evaluation and targeted intervention [2,3].

Recent advances in spinal-hip biomechanics have clarified how the hip and lumbar regions act synergistically to maintain sagittal balance, pelvic alignment, and smooth functional transitions such as standing-to-sitting movements. Disruptions in this coordination are known to influence load distribution and compensatory movement strategies, thereby affecting the onset and persistence of lower back pain (LBP) [4]. For physiotherapists, such biomechanical insights form a foundation for clinical reasoning, patient assessment, and rehabilitation planning. Hence, effective translation of biomechanical evidence into practice is a critical component of evidence-based practice (EBP) linking mechanistic understanding with patient-centered outcomes.

The integration of spinal-hip biomechanics into evidence-based physiotherapy behavior can be understood through the interaction of three interdependent domains: mechanistic understanding, clinical reasoning, and behavioral implementation. Mechanistically, coordinated motion between the lumbar spine and hip is essential for maintaining sagittal balance and efficient load transfer during functional activities; dysfunction within this kinetic chain leads to compensatory patterns, altered muscle activation, and increased mechanical stress, all of which contribute to pain persistence and movement limitation [5-7].

Clinically, physiotherapists who possess a strong biomechanical foundation are better able to identify region-specific impairments, select targeted interventions, and evaluate outcomes using objective measures that reflect spinal-hip kinematic coupling [8,9]. Behaviorally, the EBP model integrating best research evidence, clinical expertise, and patient preferences requires practitioners to actively translate biomechanical findings into individualized, function-oriented treatment strategies [10-12]. Therefore, the extent to which clinicians apply spinal-hip biomechanics research in their decision-making reflects both their EBP competence and their ability to deliver biomechanically informed, patient-centered rehabilitation.

However, despite the established relevance of spinal-hip interaction to LBP management, evidence suggests that research findings in this domain are seldom integrated into clinical decision-making. Previous studies among Saudi physiotherapists have documented generally positive attitudes toward EBP but variable levels of knowledge, skills, and practical implementation [13-14]. Commonly reported barriers include limited training in research methods, insufficient time for evidence appraisal, and inadequate institutional support [15]. Moreover, restricted use of standardized outcome measures and suboptimal adherence to guideline-based care for LBP indicate a persistent gap between available evidence and clinical application [10].

International research mirrors these trends, highlighting systemic and behavioral challenges to EBP adoption [16]. Specifically, constraints in accessing databases, low confidence in critical appraisal, and the lack of structured mentorship have been recognized as significant barriers to implementing biomechanical knowledge into practice. Collectively, this evidence underscores the need to not only assess general EBP competence but also to examine discipline-specific evidence translation, such as the integration of spinal-hip biomechanics research into physiotherapy practice.

To date, no study has explicitly investigated how Saudi physiotherapists apply empirical findings from spinal-hip biomechanics within their clinical routines. Assessing this application across the Kingdom’s 13 regions is crucial for identifying current gaps, contextual barriers, and opportunities for targeted skill development. Accordingly, this study aims to explore Saudi physiotherapists’ perceptions, use, and barriers related to integrating spinal-hip biomechanics research within evidence-based clinical practice.

## Materials and methods

Survey development

**Figure 1:**
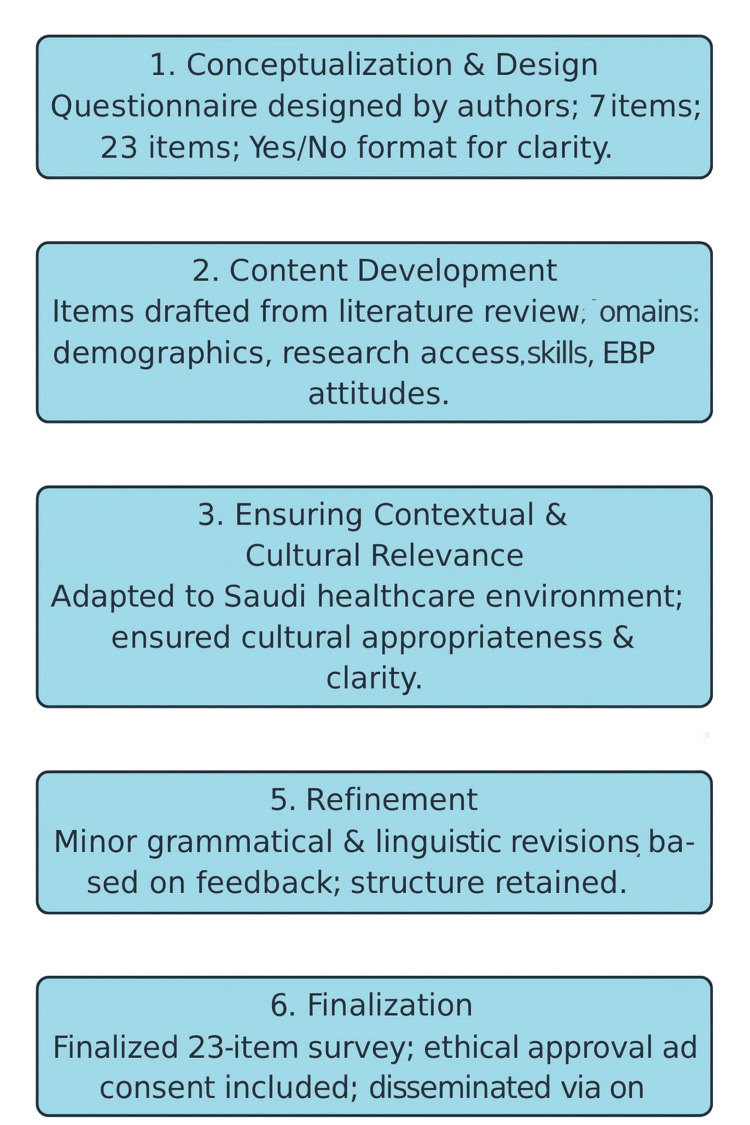
Diagram depicting the stages of constructing the study questionnaire EBP: evidence-based practice

A self-administered, cross-sectional, questionnaire-based survey was designed specifically for this study by the author (R.S.). The instrument comprised seven sections with 23 items and was structured to capture demographic details, professional research engagement, and attitudes toward integrating EBP in physiotherapy. The cross-sectional questionnaire approach is widely used in healthcare research, given its feasibility, cost-effectiveness, and ability to capture data from diverse populations within a defined period [17-18]. To ensure transparency and methodological clarity, the process of constructing the study questionnaire is presented schematically in Figure 1. This diagram serves to summarize the logical progression followed in developing the instrument, providing readers with a concise visual representation of the overall procedure and enhancing the comprehensibility of the methods employed.

Pilot testing and content validity

Before distribution, pilot testing was undertaken to evaluate the content validity, clarity, and readability of the questionnaire items. Pilot studies are considered a critical step in survey development to refine wording, ensure comprehension, and reduce potential measurement error [19-20]. A purposive sample of 10 physiotherapy practitioners from Saudi Arabia participated in the pilot, representing a range of practice contexts: orthopaedics (n = 2), outpatient clinics (n = 3), and academia (n = 5). While participants recommended minor grammatical and linguistic revisions, they confirmed the overall clarity, structure, and content validity of the instrument. However, the primary objective was to develop and preliminarily validate a context-specific instrument tailored to Saudi physiotherapists’ application of spinal-hip biomechanics research, a domain for which no pre-existing validated tool was available. Hence, adaptation from existing generic EBP scales [12] was not feasible without compromising on content specificity.

Participants and recruitment

The final version of the survey was disseminated, and 64 physiotherapy practitioners from 13 regions across Saudi Arabia completed the study. This sample size aligns with prior physiotherapy-related survey research conducted in Saudi Arabia [18], which also reported responses from 64 physiotherapists across diverse regions. The sample size for the current study was not based on an a priori power calculation; rather, it was aligned with Hasani et al. [18] to maintain methodological consistency with established research and to ensure adequate representation of physiotherapy practitioners from both clinical and academic settings. Nevertheless, the absence of a formal power analysis may limit the statistical generalizability of the findings, and this should be considered when interpreting the results. Eligible participants were required to be licensed physiotherapy practitioners working in either clinical or academic contexts, regardless of gender or nationality. Exclusion criteria included undergraduate and internship students - to safeguard data reliability - and healthcare providers from other disciplines.

The primary objective of the present study was not to evaluate or compare differences in the provision of medical care, environmental factors, or educational standards across the 13 Saudi regions. Instead, the study focused specifically on assessing physiotherapists’ awareness and application of recent spinal-hip biomechanics research and identifying barriers affecting their EBP. The inclusion of participants from 13 regions served primarily to ensure broad geographical representation of physiotherapists across the Kingdom, rather than to analyze regional healthcare or educational disparities. Therefore, regional data were captured only to reflect sample distribution and not to draw conclusions about regional variations in healthcare provision or training environments. 

Ethical considerations

The study received ethical clearance from the Najran University Research Ethics Committee (approval no. 202312-076-016107-037172; dated 27/11/2023). Informed consent was obtained electronically. All participants were provided with detailed information regarding the study objectives, the voluntary nature of the participation, and assurance of confidentiality and anonymity in compliance with international research ethics standards [21].

Data collection and distribution

The survey was hosted on a secure Google online platform. Anonymized data were accessible exclusively to the research team. The survey introduction included the consent form, study objectives, and a brief overview. To maximize reach, the survey was promoted through social media networks (Facebook, Twitter) and WhatsApp, consistent with evidence highlighting the effectiveness of digital platforms for engaging healthcare professionals in survey research [21]. The survey was open for 13 months (July 2023 - August 2024) to allow broad participation. Although social networking sites (SNS)-based recruitment may introduce some selection bias, since physiotherapists active on social platforms may be more research-engaged, this approach was necessary and appropriate given the absence of a national physiotherapy registry in Saudi Arabia. SNS has been widely validated in healthcare research as an efficient tool for reaching geographically dispersed clinicians and achieving broad national coverage.

To reduce potential bias, the survey was distributed across multiple SNS channels and professional groups to maximize demographic and clinical diversity. As the study aimed to examine physiotherapists’ application of recent spinal-hip biomechanics research rather than compare SNS users to non-users, any residual bias is unlikely to have influenced the study’s main outcomes.

Questionnaire structure

The questionnaire employed a Yes/No (YN) response format, a method used previously in Saudi physiotherapy research to capture practitioners’ knowledge and attitudes [17]. The 23 survey items encompassed several key domains relevant to clinical and research practices in physiotherapy. The first domain focused on personal details, including participants’ age, gender, and region of employment, providing essential demographic information. The second domain examined access to research databases and journals, capturing the availability of scientific resources to clinicians and researchers. The third domain explored respondents’ ability to search, analyze, critique, and synthesize scientific literature, assessing their capacity to engage with and interpret evidence. The fourth domain addressed recognition of the importance of evaluating new research findings, reflecting the value placed on integrating emerging knowledge into practice. The fifth domain focused on the implementation of novel ideas and methodologies in clinical practice, indicating the extent to which practitioners adopt innovative approaches. The sixth domain assessed awareness of the accessibility and feasibility of research tools in rehabilitation facilities, highlighting potential resource-related facilitators or barriers. Finally, the seventh domain investigated familiarity with and application of spinal measurement research, its correlation with hip biomechanics, and barriers to implementation, which constituted the largest section of the survey with 11 items and emphasized the integration of specialized biomechanical research into clinical settings. 

The survey’s design also accounted for cultural and contextual relevance within the Saudi healthcare environment, reflecting recommendations that healthcare questionnaires should be tailored to local practice settings [22,23]

Data processing

The processing of data in this study was relatively straightforward due to the structured nature of the questionnaire design. Preliminary descriptive analyses were conducted using the built-in calculator function on Google Forms, which provides automatic percentage distributions for each item response. While such built-in tools are valuable for generating basic frequency statistics, they can be limited in scope for subgroup comparisons [24]. Therefore, additional statistical computations were performed using Microsoft Excel to compare responses between male and female participants across the measured variables. To assess potential associations among categorical demographic variables, chi-square tests of independence were employed. This approach was applied to examine the relationships between age and sex, age and region, and sex and region.

To further investigate potential demographic influences, participants were stratified into five age categories: Group 1 (G1) included those aged 20-25 years, Group 2 (G2) represented 25-30 years, Group 3 (G3) comprised 30-35 years, Group 4 (G4) represented 35-40 years, and Group 5 (G5) included participants older than 40 years. Such age-based stratification is consistent with recommendations for subgroup analysis in survey research, as it allows for the identification of trends and differences that may be masked in aggregate data [25,26]. The combination of descriptive statistics with subgroup analyses enhances the interpretability and validity of survey findings by situating responses within relevant demographic contexts [27].

The analytical plan was intentionally descriptive and exploratory, consistent with the study’s aims and the binary nature of the data. As recommended by recent methodological guidance for categorical health-survey data [28], descriptive statistics and chi-square tests were the most appropriate analytic techniques for Yes/No responses and a modest sample size. All chi-square test assumptions were examined and met.

## Results

All participants consented to join the study (Table 1). Overall, male participation was higher than female participation. Regionally, Asir recorded the highest rates (14% among males and 3% among females), while the lowest rates appeared in Al-Bahah and Al-Jawf (1.6% for each gender) and in the Northern Borders (0% for males and 3% for females). In Jazan, participation reached 12.5%, represented exclusively by female participants.

**Table 1:** Percentage and frequency of participants by gender across 13 regions of Saudi Arabia (n = 64)

Region	Male, n (%)	Female, n (%)	Total, n (%)
Medina Region	3 (3.13%)	1 (1.56%)	4 (4.69%)
Asir Region	9 (14.06%)	2 (3.13%)	11 (17.19%)
Riyadh Region	4 (6.25%)	1 (1.56%)	5 (7.81%)
Al-Qassim Region	0 (0%)	4 (6.25%)	4 (6.25%)
Makkah Region	5 (7.81%)	2 (3.13%)	7 (10.94%)
Najran Region	6 (9.38%)	2 (3.13%)	8 (12.50%)
Ha’il Region	2 (3.13%)	0 (0%)	2 (3.13%)
Eastern Region	1 (1.56%)	2 (3.13%)	3 (4.69%)
Tabuk Region	5 (7.81%)	2 (3.13%)	7 (10.94%)
Jazan Region	8 (12.50%)	0 (0%)	8 (12.50%)
Al-Bahah Region	1 (1.56%)	1 (1.56%)	2 (3.13%)
Al-Jawf Region	1 (1.56%)	1 (1.56%)	2 (3.13%)
Northern Borders Region	0 (0%)	2 (3.13%)	2 (3.13%)
Total	45 (64.06%)	19 (35.94%)	64 (100%)

**Figure 2:**
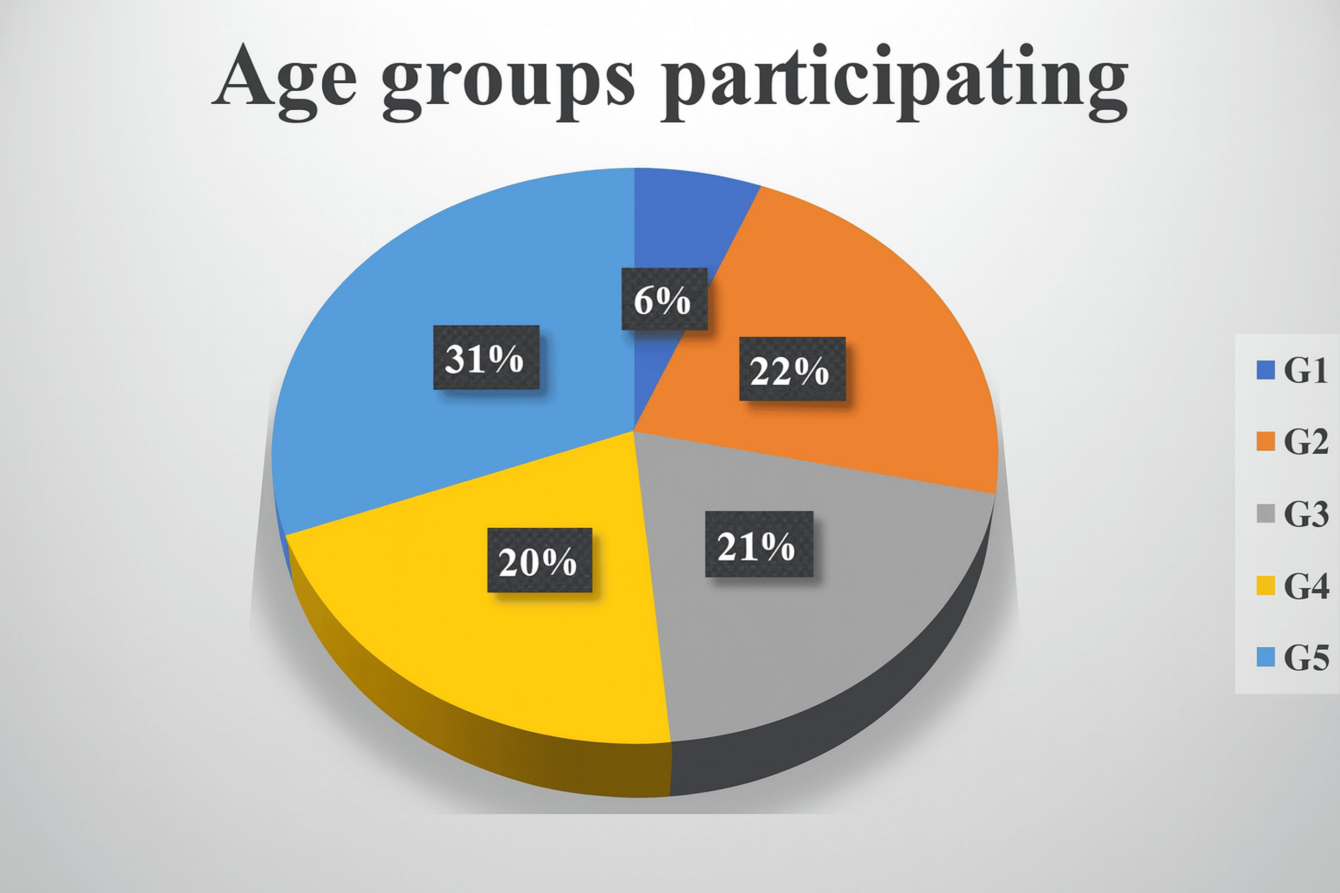
Percentage of participation by age groups across Saudi Arabia

A total of sixty-four physiotherapy practitioners were involved in this study. Among them, 31% were G5, 22% were G2, 21% were G3, 20% were G4, and 6% were G1 (Figure 2).

**Figure 3:**
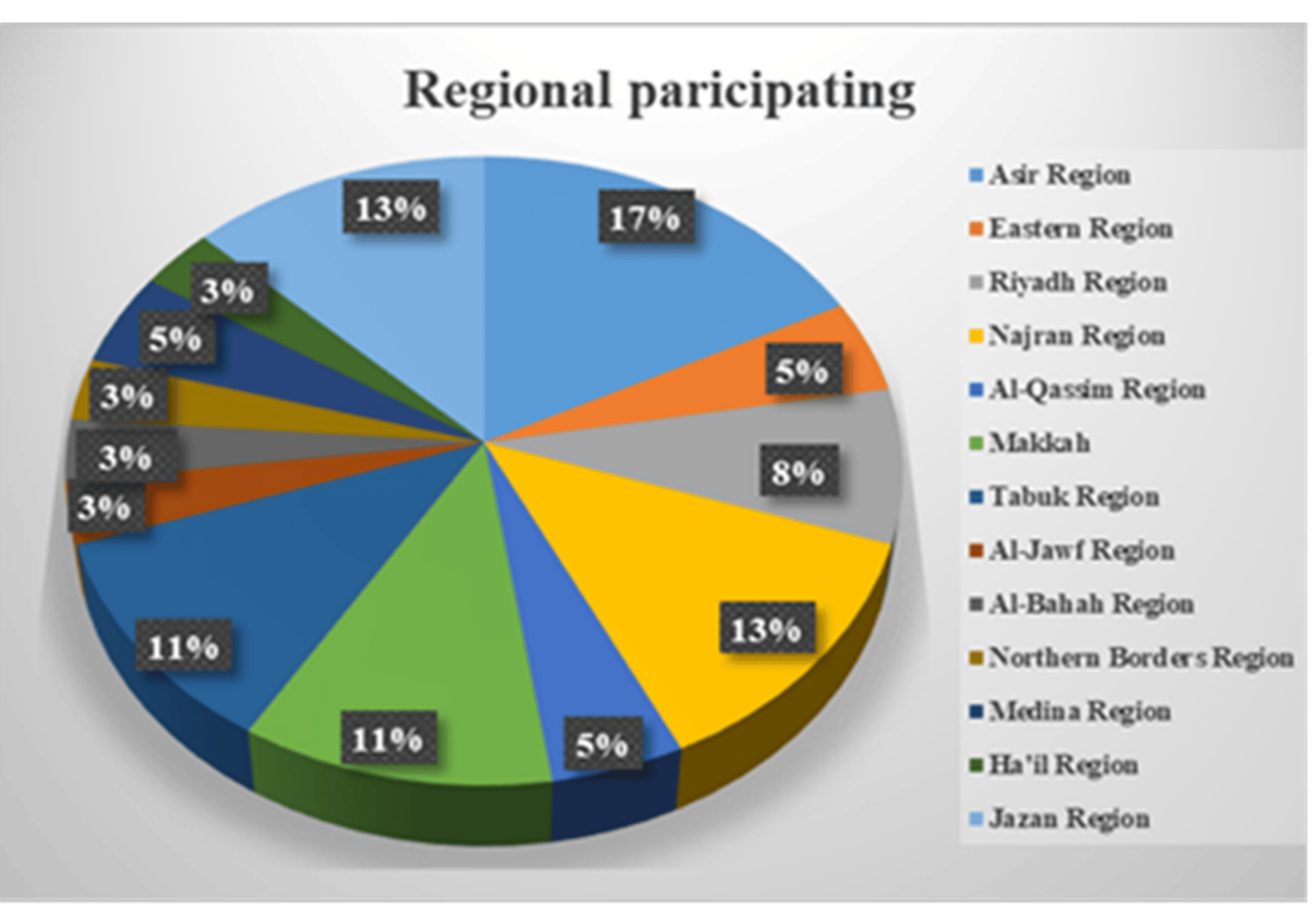
Regional participation percentage across Saudi Arabia

As illustrated in Figure 3, participation rates varied across regions. The results also indicated that respondents were drawn from all 13 regions of the Kingdom. Asir recorded the highest participation rate at 17%, followed by Najran and Jazan, each at 13%. Makkah and Tabuk ranked next with equal participation levels of 12%. Riyadh contributed 8%, while the remaining regions reported participation rates ranging from 3% to 5%.

Over 82% of males and 85% of females responded affirmatively to all items related to physiotherapy’s accessibility to medical rehabilitation research, its limitations, and the use of recent research findings as EBP to improve patient outcomes. The lowest affirmative response rates were associated with two items: the availability of institutional facilities to support research needs (such as databases) and the need for training on new physiotherapy devices (Table 2). Notably, female respondents reported higher positive response rates than males across all items except one: the ability to read, critique, and summarize scientific research, where females scored 7% lower than males.

**Table 2:** Percentage of male and female physiotherapists with access to medical rehabilitation facilities and application of EBP EBP: evidence-based practice

Items	Male, n (%)		Female, n (%)	
	Yes	No	Yes	No
Does your institution provide you with free access to the latest research and medical rehabilitation databases?	28 (66.7%)	14 (33.3%)	18 (81.8%)	4 (18.2%)
Do you have the skills to search for new developments and scientific research?	38 (90.5%)	4 (9.5%)	20 (90.9%)	2 (9.1%)
Do you have the skills to read, criticize, and summarize scientific research in your field?	35 (83.3%)	7 (16.7%)	17 (77.3%)	5 (22.7%)
Do you think that periodic access to medical rehabilitation research is inevitable or limited to the basic information you have learned?	36 (85.7%)	6 (14.3%)	20 (90.9%)	2 (9.1%)
Do you, as a physiotherapist, apply research findings and recommendations in patient assessment protocols?	35 (83.3%)	7 (16.7%)	20 (90.9%)	2 (9.1%)
Do you think the practical application of these recommendations and outputs will increase your confidence and provide a quality service to the patient?	35 (83.3%)	7 (16.7%)	18 (81.8%)	4 (18.2%)
Is there a limitation to obtaining modern measuring devices used in scientific research and their availability in your physiotherapy clinics?	40 (95.2%)	2 (4.8%)	21 (95.5%)	1 (4.5%)
Is there a need for training in scientific methods and modern instruments of measurement?	30 (71.4%)	12 (28.6%)	16 (72.7%)	6 (27.3%)

The assessment of spinal-dominant motion areas and their measurement methods indicated that affirmative responses were consistently lower than negative responses across all items for both male and female participants, except for a single item in which males recorded a 59.5% affirmative rate (Table 3).

**Table 3:** Use of EBP to guide spinal assessment choice and tool accessibility by gender EBP: evidence-based practice

Items	Male, n (%)		Female, n (%)	
	Yes	No	Yes	No
It is known that the dominant motion areas in the spine are the lumbar, cervical, and then the thoracic spine, as well as it is also known that the specialist measures the ROM of these areas as one component previously. Have you read recent studies that measure the three regions individually?	18 (42.9%)	24 (57.1%)	8 (36.4%)	14 (63.6%)
Do you measure the lumbar, the thoracic, and the cervical spine separately?	20 (47.6%)	22 (52.4%)	9 (40.9%)	13 (59.1%)
Have recent studies recommended to measure the individual measurement of these regions?	20 (47.6%)	22 (52.4%)	9 (40.9%)	13 (59.1%)
Have you read studies that divided the lumbar spine into two regions (the upper and lower lumbar spines)?	15 (35.7%)	27 (64.3%)	5 (22.7%)	17 (77.3%)
Do you know that the lower lumbar spine contributes more than twice as much motion in flexion tasks as the upper lumbar, increasing the risk of injuries and disc damage?	25 (59.5%)	17 (40.5%)	10 (45.5%)	12 (54.5%)
Do you, as a physiotherapist, rely on calculating regional velocities to comprehend regional motion behaviors and judge if they are normal or abnormal?	21 (50.0%)	21 (50.0%)	6 (27.3%)	16 (72.7%)
Have you found a study that has concluded that the measurement of lumbar spine flexion should not be used as a sole test in spinal assessment, but that several tasks must be added to produce accurate measurements for spinal evaluation protocols?	11 (26.2%)	31 (73.8%)	9 (40.9%)	11 (50.0%)
Have you read a study that claimed that measuring the lumbar region as one unit rather than two distinct sections would exclude some crucial mechanical data?	10 (23.8%)	32 (76.2%)	3 (13.6%)	19 (86.4%)

Two principal hindrances were identified as limiting the translation of recent findings into EBP: the scarcity of educational and academic institutions and the restricted capacity to accept, implement, and license advanced research. These impediments were reported by 33.3% of males and 41% of females. Moreover, the limited contribution of professional associations in physiotherapy was reported by 23.8% of males and 4.5% of females, while the absence of institutional frameworks providing patient services was noted by 9.5% of males and 13.6% of females (Table 4).

**Table 4:** The greatest challenge to applying recent research findings as EBP by gender EBP: evidence-based practice

Items	Male, n (%)	Female, n (%)
Limited role of scientific associations in physiotherapy	10 (23.8%)	1 (4.5%)
Lack of educational and academic institutions	14 (33.3%)	9 (40.9%)
Inability to accept, utilize, and license advanced research	14 (33.4%)	9 (40.9%)
Absence of institutional structure offering patient services	4 (9.5%)	3 (13.6%)

As shown in Table 5, the chi-square tests indicated a significant association between age and sex (χ² = 12.84, df = 4, p = 0.012) and between sex and region (χ² = 26.31, df = 12, p = 0.010), suggesting that age distributions differed by sex and that the representation of males and females varied across regions. In contrast, the association between age and region was not significant.

**Table 5:** Chi-square tests for age, sex, and region reporting degrees of freedom, p-values, and significance for each comparison Chi-square test of independence; p<0.05 was considered statistically significant

Comparison	Chi-square	Df	P-value	Significance (p<0.05)
Age vs. sex	12.83653	4	0.012103	Yes
Age vs. region	42.22708	48	0.707403	No
Sex vs. region	26.31219	12	0.009693	Yes

## Discussion

This study examined physiotherapists in the Kingdom of Saudi Arabia regarding their understanding of contemporary biomechanical research on the spinal-hip relationship, their ability to integrate this knowledge into clinical decision-making, and the subsequent influence on their confidence during patient interactions. The study further assessed the extent to which these findings were implemented in practice and explored barriers that impede the integration of contemporary biomechanical evidence into EBP. To our knowledge, no prior research has examined physiotherapists’ use of recent spine-hip biomechanical findings, making this study unique and difficult to compare directly with earlier work.

Overall, the findings indicate that physiotherapists in Saudi Arabia generally have access to research resources, institutional facilities, and the basic capacity to translate knowledge into practice. Between 66.7% and 95.5% of participants reported opportunities to engage with EBP, and most expressed favorable attitudes toward evidence-based physiotherapy. These findings are consistent with national research highlighting positive attitudes toward EBP among healthcare professionals [17-20,29,30] and mirror international trends demonstrating that physiotherapists widely endorse the value of research-informed practice [31].

Despite these positive attitudes, the implementation of research remained varied. Although 86% of participants reported applying recent research in clinical settings and 85.5% observed improved outcomes following evidence-based interventions, knowledge of advanced spinal biomechanics was relatively limited. Only 42% had previously engaged with studies analyzing spinal regions independently, and merely one-third were familiar with evidence differentiating upper and lower lumbar segments. While half recognized the greater mobility and injury susceptibility of the lower lumbar spine, fewer translated this knowledge into targeted clinical assessments or treatment approaches. Similarly, only 51% reported assessing lumbar-hip range of motion, and only 42% incorporated velocity analysis into evaluations despite evidence supporting its diagnostic and functional relevance.

Additionally, only one-third of physiotherapists incorporated functional tasks such as sit-to-stand transitions into spinal assessments, despite prior research demonstrating the value of functional movement analysis in identifying load-sharing patterns and compensatory strategies [32]. This disconnect between theoretical awareness and practical application is consistent with the broader literature on the research-practice gap in physiotherapy [14,33] and highlights the need for targeted training in biomechanics translation.

Demographically, the study categorized participants into five age clusters to evaluate potential generational differences in EBP adoption. Chi-square analyses revealed a significant association between age and sex, indicating uneven distribution across groups (χ² = 12.84, p = 0.012). Sex and region were also significantly associated (χ² = 26.31, p = 0.010), suggesting regional variation in gender representation. In contrast, age and region showed no significant relationship (χ² = 42.23, p = 0.707). Collectively, these findings suggest that sex appeared to influence certain patterns in the sample. However, given the modest sample size and uneven distribution across regions, these associations should be interpreted cautiously. In the revised manuscript, claims about demographic influences have been framed as exploratory rather than definitive.

Institutional access to databases emerged as an important facilitator of EBP engagement. Approximately two-thirds of institutions provided complimentary access to scientific databases, supporting clinicians’ information-seeking behaviors, while nearly one-third lacked such access, limiting their ability to remain current with evolving evidence. A significant majority (90.6%) reported the ability to search for and retrieve articles, and 80% could read, critique, and summarize research findings. Nonetheless, 13% believed that textbooks alone were sufficient, likely reflecting insufficient exposure to formal EBP training within academic programs.

Despite a strong research interest, participants identified several systemic barriers that affected their ability to implement contemporary spine-hip biomechanics findings; 37% attributed challenges to institutional and educational systems, 35% cited the absence of formal mechanisms for endorsing or licensing new physiotherapy research, 18% emphasized the limited influence of professional associations, and 11% noted resistance to EBP within healthcare delivery systems. These observations align with earlier reports describing limited critical-appraisal training, uneven access to journals, insufficient protected research time, and minimal organizational support within physiotherapy settings [10,31].

The use of SNS for recruitment may have introduced selection bias, as clinicians more active on SNS may be more inclined toward professional engagement. However, SNS-based recruitment is widely used in contemporary physiotherapy and health research and is recognized as efficient for accessing geographically dispersed clinician populations [34-37]. In this context, SNS platforms allowed access to physiotherapists across 13 regions, supporting diverse representation in the absence of a national physiotherapy registry.

This study has several limitations. The sample size was relatively small (n = 64), limiting generalizability, and uneven regional representation may have introduced bias. Moreover, reliance on self-reported measures may have inflated estimates of EBP engagement, as actual clinical behaviors were not observed. Additionally, the cross-sectional design limits causal interpretation.

Despite these limitations, this study provides foundational evidence on physiotherapists’ engagement with advanced spinal-hip biomechanics research and highlights critical gaps between awareness and practical application. The findings underscore the need for targeted educational initiatives, improved institutional support, and strengthened professional development pathways to enhance knowledge translation and optimize patient care outcomes.

## Conclusions

This study provides the first nationwide insights into Saudi physiotherapists’ engagement with contemporary spinal-hip biomechanics research. While attitudes toward EBP were positive and access to research resources was generally available, the practical application of advanced biomechanical concepts remained limited. Systemic barriers - including variable institutional support and inconsistent access to scientific databases - further constrained evidence integration. Strengthening targeted education, professional development, and institutional EBP infrastructure is essential to improve knowledge translation and enhance patient outcomes. Future research with larger sample sizes is needed to validate and expand upon these findings. The findings highlight the need to strengthen physiotherapists’ training in contemporary spinal-hip biomechanics to ensure more accurate assessment and targeted intervention. Improving access to research databases, providing structured EBP education, and integrating functional movement analysis into routine practice may enhance clinical decision-making and patient outcomes. Institutions and professional bodies should support ongoing professional development to facilitate the translation of emerging biomechanical evidence into everyday clinical care.
